# Human cytomegalovirus seroprevalence and titres in solid organ transplant recipients and transplant donors in Seoul, South Korea

**DOI:** 10.1186/s12879-019-4607-x

**Published:** 2019-11-08

**Authors:** Yeonju La, Da Eun Kwon, Seul Gi Yoo, Kyoung Hwa Lee, Sang Hoon Han, Yong Goo Song

**Affiliations:** Department of Internal Medicine, Division of Infectious Disease, Yonsei University College of Medicine, Seoul, Republic of Korea

**Keywords:** Age, Human cytomegalovirus, Seroprevalence, Solid organ transplantation

## Abstract

**Background:**

Human cytomegalovirus (HCMV) can cause poor outcomes in solid organ transplant (SOT) recipients; moreover, it is associated with cardiovascular diseases (CVD) in the general population. Accordingly, anti-HCMV immunoglobulin G (IgG) seroepidemiology may be useful in identifying the risk of post-SOT HCMV infection or disease as well as immunosenescence or CVD. However, HCMV seroprevalence and titre have not been fully evaluated with regard to age distribution or compared between SOT recipients and healthy individuals in South Korea.

**Methods:**

We retrospectively retrieved all unduplicated anti-HCMV IgG results of individuals aged > 1 year evaluated between July 2006 and November 2017 at Severance Hospital in Seoul. The cohort, excluding haematopoietic stem cell transplant recipients and subjects with equivocal values, included 2184 SOT recipients and 3015 healthy transplant donors. All IgG results in the SOT recipients were measured during the pre-transplant period.

**Results:**

The overall IgG seroprevalence and titres were significantly higher among SOT recipients than among healthy donors (98.7% vs. 88.6%, *p* < 0.001, and 64.7 ± 44.3 vs. 49.8 ± 20.6 arbitrary units/mL, *p* < 0.001, respectively). The lowest seropositive rate in the SOT group was observed in recipients aged between 11 and 15 years (70.6%). The frequency of seropositivity among adults aged ≥41 years increased to ≥90% in SOT recipients and healthy donors. Age was independently associated with higher HCMV seroprevalence (41–60 years, OR, 76.4, 95% CI, 24.5–238.9, *p* < 0.001; ≥ 61 years, OR, 4.4, 95% CI, 1.3–14.9, *p* < 0.001, compared to ≤40 years). The healthy donor group had an independently low HCMV seropositive rate (OR, 0.1, 95% CI, 0.1–0.2, *p* < 0.001).

**Conclusions:**

HCMV seropositivity was the lowest among school-aged children and adolescents. IgG testing revealed an intermediate serostatus risk of post-transplant HCMV infection and disease for most adult SOT recipients in South Korea.

## Background

The genomic DNA of human cytomegalovirus (HCMV) can be incorporated into the host chromosome after primary acquisition, usually in early life, and be retained in a latent form throughout life [1–4]. The complicated mechanisms involved in the latent-to-lytic switch can induce temporary or sustained HCMV replication, leading to active cytolytic inflammation [1, 3, 4]. In both immunocompetent and immunocompromised patients, reactivation of latent HCMV can result in direct tissue damage that causes end organ disease [1, 5–7]. In addition, the indirect immunomodulatory effects caused by the reactivated HCMV may produce detrimental outcomes, such as increased mortality and graft dysfunction or failure and rejection, in recipients of solid organ transplantation (SOT) [8, 9]. Moreover, the persistent and excessive activation of the HCMV-specific cell-mediated immune response and the consequent immune exhaustion or accelerated immunosenescence in the non-immunocompromised population could predispose these individuals to chronic inflammatory diseases, such as cardiovascular diseases (CVDs), as well as aging [10–13].

The profound clinical influence of HCMV on personal and public health makes it reasonable to acquire detailed information regarding the number of individuals that have asymptomatic HCMV in a certain region [14]. Surveillance for HCMV seroprevalence, which is assessed using an anti-HCMV immunoglobulin G (IgG) test, may be epidemiologically important as a means of predicting various related chronic diseases, particularly in older subjects, as global societies age [11, 12, 15]. Additionally, the pre-transplant HCMV serostatus in donors and recipients is an important factor predicting post-transplant HCMV infection and disease; moreover, it provides information that would be useful in developing individually tailored anti-HCMV preventive strategies for SOT recipients [1, 3, 9, 16, 17].

The anti-HCMV IgG positive rates vary widely by era, geographic distribution, age group, and population characteristics [18, 19]. In 2019, Zuhair et al. estimated the mean HCMV seroprevalence in the global general population to be 83% (95% uncertainty interval, 78–88%), ranging from 90% (85–94%) in the Eastern Mediterranean region to 66% (56–74%) in Europe, among the six regions demarcated by the World Health Organization (WHO) [14]. The national burden of HCMV also varies between 18% in Canada and 100% in Egypt, Iran, Thailand, and Turkey [14]. However, no report, to our knowledge, has evaluated HCMV seroepidemiology across all ages of a general population, among women of reproductive age, or among SOT recipients in South Korea, which has experienced rapid socioeconomic development and advances in public health in recent years [14, 20].

Therefore, this study aimed to evaluate the recent HCMV seroprevalence and anti-HCMV IgG titres among SOT recipients, particularly according to age distribution, in Seoul, South Korea.

## Methods

### Study design and population

For the retrospective cohort study, we retrieved all unduplicated anti-HCMV IgG results and IgG titres for tests performed between July 2006 and November 2017 from the electronic medical records of Severance Hospital, a university-affiliated tertiary care centre in Seoul, South Korea. Infants (*n* = 488) were excluded, because they could possess maternal anti-HCMV IgG for a year after birth [21]. After the further deletion of 34 equivocal results, we stratified the total cohort into two groups of 2184 SOT recipients and 3015 healthy transplant donors who underwent this screening for organ donation, irrespective of the implementation of donation surgery. The SOT recipients group included all recipients who received transplants from living or deceased donors. Our cohort did not include SOT recipients had received haematopoietic stem cell transplantation (HSCT) before and after SOT or re-transplantation or multiple-organ transplantation or human immunodeficiency virus-infected individuals. For all SOT recipients, anti-HCMV IgG values were evaluated during the pre-transplant period within 3 months of SOT. The ages at the time of testing, anti-HCMV IgG titres, and transplant organs were also recorded. Patient ages were grouped in 5-year intervals up to 40 years of age and, then, in 20-year intervals. The study was approved by the institution review board in Gangnam Severance Hospital, which waived the requirement for informed consent because of retrospective data collection without personally identifiable information (IRB approval No: 3–2018-0139).

### Measurement of anti-human cytomegalovirus IgG antibody

Serum samples were automatically subjected to quantitative anti-HCMV IgG testing using an enzyme-linked fluorescent immunoassay (VIDAS®, bioMérieux Cop., Marcy-l’Etoile, Lyon, France). Antigens from the HCMV AD169 laboratory strain were used as the positive control. The antibody titre is expressed in arbitrary units (aU)/mL. Qualitatively, the results were reported as positive, equivocal, and negative if the titres were ≥ 6, 4 to < 6, and < 4 aU/mL, respectively. The results of anti-HCMV IgG tests in the analyses were categorised as positive or negative.

### Statistical analyses

We used the independent two-sample t-test and the chi-square test or Fisher’s exact test, respectively, to examine differences in continuous and nominal variables between SOT recipients and healthy transplant donors. The post-hoc analyses in the nominal variables were performed by adjusted standardised residuals to control for type I error inflation (adjusted *p* value). An analysis of variance (ANOVA) with the Bonferroni post-hoc test was used for multiple comparisons of IgG titres between transplant organs. Multivariate logistic regression analyses were performed to identify the independent effects of age groups and sex on HCMV seropositivity. Data are expressed as numbers (percentages) or means ± standard deviations or odds ratio (OR) (95% confidence intervals [CI]). SAS version 9.3 (SAS Institute Inc., Cary, NC, USA) was used for the statistical analyses. A two-tailed *p*-value ≤0.05 was considered statistically significant.

## Results

### HCMV IgG results from SOT recipients and healthy transplant donors

The overall anti-HCMV IgG positivity rate was 92.8% in the total of 5199 subjects. The HCMV seropositivity in males was not significantly different from that in females among SOT recipients (99.8% [*n* = 1354] vs. 98.4% [*n* = 830], *p* = 0.564). However, among healthy transplant donors, males had a significantly lower HCMV seropositive rate (84.1% [*n* = 1681] vs. 86.8% [*n* = 1334], *p* = 0.039). The frequency of anti-HCMV IgG positivity in all ages was significantly higher among SOT recipients than among healthy transplant donors (98.7% vs. 88.6%, *p* < 0.001) (Table 1).

**Table 1:** HCMV IgG seroprevalence in solid organ transplant recipients and healthy transplant donors by age distribution

Age (years)	Anti-HCMV IgG	SOT recipients (*n* = 2184)	Healthy transplant donors (*n* = 3015)	*p*-value
1–5	Positive	12 (80.0)	0 (0)	N/A
	Negative	3 (20.0)	0 (0)	
6–10	Positive	9 (75.0)	0 (0)	N/A
	Negative	3 (25.0)	0 (0)	
11–15	Positive	12 (70.6)	0 (0)	N/A
	Negative	5 (29.4)	0 (0)	
16–20	Positive	41 (93.2)	46 (54.8)	<0.001
	Negative	3 (6.8)	38 (45.2)	
21–25	Positive	54 (90.0)	162 (63.3)	<0.001
	Negative	6 (10.0)	94 (36.7)	
26–30	Positive	114 (96.6)	262 (78.9)	<0.001
	Negative	4 (3.4)	70 (21.1)	
31–35	Positive	133 (99.3)	307 (86.5)	<0.001
	Negative	1 (0.7)	48 (13.5)	
36–40	Positive	215 (99.1)	229 (89.8)	<0.001
	Negative	2 (0.9)	26 (10.2)	
41–60	Positive	1287 (99.8)	1088 (94.3)	<0.001
	Negative	2 (0.2)	66 (5.7)	
≥ 61	Positive	278 (100)	576 (99.5)	0.555
	Negative	0 (0)	3 (0.5)	
Total	Positive	2155 (98.7)	2670 (88.6)	<0.001
	Negative	29 (1.3)	345 (11.4)	

Data are expressed as number (percent). Abbreviations: *HCMV* Human cytomegalovirus, *IgG* immunoglobulin G, *N/A* not available, *SOT* solid organ transplantation

### HCMV seroprevalence according to age groups

There were few subjects aged ≤15 years in the SOT recipients group (44/2184, 2.0%) and none in the group of 3015 healthy transplant donors. The proportion of anti-HCMV IgG positive results was extremely high among SOT recipients aged ≥31 years and among healthy transplant donors aged ≥61 years. In contrast, HCMV seropositive rates were the lowest among those between 11 and 15 years of age in the SOT recipients group (70.6%) and those between 16 and 20 years of age among the healthy transplant donors (54.8%). The HCMV seropositivity in the healthy donors continuously increased with age from 54.8% in those aged between 16 and 20 years to 99.5% in those ≥61 years of age (Table 1).

SOT recipients had significantly higher rates of seropositivity, compared to healthy transplant donors, among those between 16 to 20 (93.2% vs. 54.8%, *p* < 0.001), 21 to 25 (90.0% vs. 63.3% *p* < 0.001), 26 to 30 (96.6% vs. 78.9%, *p* < 0.001), 31 to 35 (99.3% vs. 86.5%, *p* < 0.001), 36 to 40 (99.1% vs. 89.8%, *p* < 0.001) and 41 to 60 years of age (99.8% vs. 94.3%, *p* < 0.001). The very low HCMV seropositive rates in both SOT recipients (0%) and healthy transplant donors (0.5%) among those aged ≥61 years were not significantly different (*p* = 0.555) (Table 1).

### Anti-HCMV IgG titres in SOT recipients and healthy transplant donors

The overall HCMV IgG titre in subjects showing HCMV seropositivity was significantly higher among SOT recipients (*n* = 2155) than among healthy transplant donors (*n* = 2670; 64.7 ± 44.3 vs. 49.8 ± 20.6 aU/mL; *p* < 0.001). In an analysis according to age distribution, SOT recipients had significantly higher HCMV IgG titres at all analysable ages than the healthy transplant donors (Table 2). ANOVA testing revealed that age subgroups had significantly different HCMV IgG titres in both SOT recipients and healthy donors (overall *p* < 0.001).

**Table 2:** Anti-HCMV IgG titre between solid organ transplant recipients and healthy transplant donors showing HCMV seropositivity by age distribution

Age (years)	Anti-HCMV IgG titre (aU/mL)		*p-*value
	SOT recipients (*n* = 2155)	Healthy transplant donors (*n* = 2670)	
16–20	56.9 ± 35.6	37.6 ± 12.9	<0.001
21–25	60.1 ± 43.9	40.6 ± 26.8	<0.001
26–30	55.0 ± 38.8	42.9 ± 30.8	<0.001
31–35	54.6 ± 27.8	45.3 ± 27.6	<0.001
36–40	64.7 ± 37.1	48.6 ± 29.5	<0.001
41–60	67.8 ± 45.3	52.1 ± 13.4	<0.001
≥ 61	70.4 ± 52.5	55.4 ± 30.1	<0.001
Total	64.7 ± 44.3	49.8 ± 20.6	<0.001

Data are expressed as mean ± standard deviation

### HCMV seroprevalence and titres according to transplant organs

The kidney (*n* = 1436, 65.8%) was most common transplant organ in this cohort. Liver transplant (LT, *n* = 579) and lung transplant (LTx, *n* = 69) recipients had significantly higher mean ages, compared to kidney transplant (KT) and heart transplant (HT) (*n* = 100) recipients (*p* < 0.001). In all age groups, overall anti-HCMV IgG titres among LT (80.1 ± 54.5 aU/mL) and LTx (84.3 ± 68.3 aU/mL) recipients were significantly higher than those among KT (59.4 ± 36.1 aU/mL) and HT (58.1 ± 46.9 aU/mL) recipients (*p* < 0.001). In addition, seropositive rates in all age groups were significantly higher in LT (99.7%) and LTx (100%) recipients than in KT (98.3%) and HT (97.0%) recipients (*p* = 0.024). However, the HCMV IgG positive rates and titres were similar among the four-transplant organs in SOT recipients aged ≤40 years (Table 3)

**Table 3:** HCMV seropositive rates and anti-HCMV IgG titre by transplant organs in solid organ transplant recipients

Age (years)	Kidney	Liver	Lung	Heart	*p-*value
	(*n* = 1436)	(*n* = 579)	(*n* = 69)	(*n* = 100)	
≤ 40					
Seropositive	478 (95.2)	57 (98.3)	15 (100)	34 (94.4)	0.716
Titre (aU/mL)	57.7 ± 36.6	65.3 ± 42.6	69.5 ± 44.4	51.1 ± 27.0	0.184
41–60					
Seropositive	801 (100)	400 (99.8)	33 (100)	47 (97.9)	0.073
Titre (aU/mL)	60.7 ± 34.6^*†^	81.3 ± 55.5^*^	88.5 ± 72.3^†^	66.4 ± 60.7	0.001
≥ 61					
Seropositive	133 (100)	120 (100)	21 (100)	16 (100)	―
Titre (aU/mL)	59.9 ± 44.2^*^	82.9 ± 56.3^*^	90.3 ± 80.5	48.8 ± 27.4	0.001
Total					
Age, year	44.9 ± 12.4^*†^	52.3 ± 12.0^*‡^	50.2 ± 13.0^†§^	44.3 ± 17.3^‡§^	<0.001
Seropositive	1412 (98.3)	577 (99.7)^a^	69 (100.0)^a^	97 (97.0)	0.024
Titre (aU/mL)	59.4 ± 36.1^*†^	80.1 ± 54.5^*‡^	84.3 ± 68.3^†§^	58.1 ± 46.9^‡§^	<0.001

Data are expressed as number (percent) or mean ± standard deviation. ^*†‡§^*p*-value <0.05 between two groups in post-hoc tests by Bonferroni correction after ANOVA. ^a^adjusted *p* < 0.05 between two groups using post-hoc analyses based on adjusted standardised residuals. Abbreviation: aU, arbitrary unit

### Regression analyses for the effect of age and sex on HCMV seropositivity

Older age (reference ≤40 years; 41–60 years: OR, 76.42, 95% CI, 24.45–238.85, *p* < 0.001 and ≥ 61 years: OR, 4.44, 95% CI, 1.32–14.91, *p* < 0.001) was an independent factor associated with a higher HCMV IgG positive rate. Healthy subjects were independently associated with a lower HCMV seropositive rate (OR: 0.12, 95% CI: 0.08–0.17, *p* < 0.001; Table 4).

**Table 4:** Multivariate logistic regression analyses to evaluate the factors associated with HCMV IgG seropositivity

Variables	OR	95% CI	*p-*value
Age, years			
≤ 40	Ref.	Ref.	Ref.
41–60	76.42	24.45–238.85	<0.001
≥ 61	4.44	1.32–14.91	0.016
Sex, male	1.33	0.98–1.64	0.078
Healthy transplant donor	0.12	0.08–0.17	<0.001

Abbreviations: *CI* confidence interval, *OR* odds ratio, *Ref* reference

## Discussion

This study reports a very high HCMV seroprevalence in Seoul, South Korea, a developed country with a high socioeconomic status and well-organised public health system [20, 22]. As a result, our data may suggest high proportions of both seropositive donors and recipients (D+/R+), which is considered an usual intermediate risk for post-transplant HCMV infection and/or disease via the reactivation of a latent virus [9, 16, 23]. Our overall HCMV IgG seropositive rate is higher than the upper seroprevalence value (88%) of the 95% uncertainty interval in the worldwide general population, based on a recent meta-regression-based estimation [14]. Seronegative individuals were extremely rare among those aged ≥31 years in the SOT recipients. In addition, HCMV seropositive rates and titres were generally proportional to age, except for teenagers. As HCMV may be horizontally transmitted by intimate contact, mainly by hand contact, the lowest seroprevalence observed in subjects between 11 to 15 years of age could be attributed to primary acquisition of HCMV at adolescence owing to improved hygiene [24, 25].

These analyses also revealed high HCMV IgG titres in the elderly population among SOT recipients and healthy subjects. A high HCMV IgG titre and persistent immune reactivation caused by an inflation in the population of long-lived, non-classical HCMV-specific effector memory CD8+ T lymphocytes have been associated with chronic inflammatory diseases, including atherosclerosis, stroke, and coronary artery disease [3, 4, 11, 26–28]. Therefore, the findings of high seropositivity and IgG titres in elderly individuals might suggest the need for further evaluation to prevent HCMV reactivation in a specific population, regardless of their immunocompromised status, as this approach could reduce the morbidity and mortality associated with inflammatory vascular diseases.

Despite the international distribution of HCMV, seropositivity rates around the world vary widely from 18 to 100%, according to geographical location, ethnicity, and specific subpopulation features [14, 29–32]. In a recent study by Li et al., stratification of serological profiles by age group revealed a very high IgG positive rate (97%) even among young individuals (0–14 years), in contrast to our data [17]. A study conducted in the Netherlands, in 2006 to 2007, reported that non-western individuals (76.7%) had a considerably higher seroprevalence than native Dutch and western individuals (41.5%) [31]. In general, the very high HCMV seroprevalence observed in South Korea is similar to that reported in the WHO Eastern Mediterranean region, rather than in the European region or the Americas [14, 29, 31, 32]. The different breastfeeding rates and HCMV IgM or IgG seropositive rates of women of reproductive age could contribute to the varied seroprevalence of HCMV between countries or regions, because mother-to-infant vertical transmission may have a major impact on global epidemiology of HCMV [14, 33, 34].

In a SOT setting reported in Hungary, living organ donors were found to have an HCMV seroprevalence of 85% [35]. However, a detailed analysis of the pre-transplant HCMV IgG seropositivity rates and titres among SOT recipients according to age groups or transplant organs as well as in contrast to healthy subjects was not fully reported. In our study, SOT recipients had higher HCMV IgG seropositive rates and titres relative to healthy transplant donors. In addition, the healthy individuals were independently associated with significantly lower HCMV IgG seroprevalence in a logistic regression model. Patients who need SOT have higher HCMV seroprevalence and, therefore, exhibit a higher risk for post-transplant complications associated with HCMV than patients who do not need SOT. Therefore, pre-transplant anti-HCMV IgG assessments are clinically significant in predicting the development of post-transplant HCMV infection and/or disease [9, 16]. In addition, the HCMV serostatus can be clinically useful to stratify the risk groups for post-transplant HCMV reactivation and guide precise HCMV preventive strategies [9, 16].

It would be informative to reveal that patients who received SOT in the end stage organ disease state have higher HCMV IgG seropositive rates and titres compared to heathy individuals. This finding might be associated with asymptomatic intermittent HCMV replication and immune boosting against HCMV in chronic end stage medical diseases as well as polyclonal immune activation through pro-inflammatory cytokine release upon fulminant acute organ failure leading to SOT [36–39].

This retrospective study has several limitations. We were unable to perform further subgroup analyses according to various underlying morbidities and other immunosuppressive conditions, such as HSCT or haematologic malignancies with severe prolonged neutropenia. In addition, the study design did not allow the serial testing of anti-HCMV IgG titres in SOT recipients at the post-transplant period and subsequent evaluation of the dynamic change of HCMV IgG titres with recovery of transplanted organ function. Moreover, HCMV seroprevalence may differ according to study population, city, and region.

Despite these limitations, this study is the first, to our knowledge, to report a difference in the qualitative and quantitative anti-HCMV IgG findings between SOT recipients and healthy individuals according to serial age groups as well as transplanted organs in SOT recipients.

## Conclusion

The overall HCMV seropositive rate in South Korea was as high as 90% among both SOT recipients and healthy individuals. Furthermore, the SOT recipients had higher HCMV seropositive rates and IgG titres than the healthy donors. Increased age was an independent factor for high HCMV seropositivity. A large-scale multicentre or regional serosurveillance in the general population as well as among immunocompromised patients who harbour high risks of HCMV infection and life-threatening end-organ diseases could be helpful.

## Data Availability

The datasets used and/or analysed during the current study are available from the corresponding author on reasonable request.

## Abbreviations

aU: Arbitrary unit
CVD: Cardiovascular disease
HCMV: Human cytomegalovirus
HSCT: Haematopoietic stem cell transplantation
HT: Heart transplantation
Ig: Immunoglobulin
KT: Kidney transplantation
LT: Liver transplantation
LTx: Lung transplantation
SOT: Solid organ transplantation
